# CA724 Predicts Tumor Regression Grade in Locally Advanced Gastric Cancer Patients with Neoadjuvant Chemotherapy

**DOI:** 10.7150/jca.60694

**Published:** 2021-09-03

**Authors:** Yilin Tong, Yanmei Zhu, Yan Zhao, Chengyao Jiang, Wentao Wang, Zexing Shan, Fan Sun, Dong Liu, Jianjun Zhang

**Affiliations:** 1 Department of Gastric Surgery, Liaoning Cancer Hospital and Institute, Cancer Hospital of China Medical University, Shenyang, China.; 2 Department of Pathology, Liaoning Cancer Hospital and Institute, Cancer Hospital of China Medical University, Shenyang, China.

**Keywords:** Gastric cancer, Tumor regression grade, Neoadjuvant therapy, Tumor marker, CA724

## Abstract

**Purpose:** Tumor regression grade (TRG) is widely used to evaluate the efficacy of neoadjuvant chemotherapy (NCT) and it is related to many clinicopathological factors. However, whether TRG can be predicted by clinical characteristics is unknown.

**Methods:** 141 locally advanced gastric cancer (GC) patients who underwent NCT and curative operation were retrospectively analyzed. TRG is reevaluated according to the CAP guideline. The values of CA199, CA125 and CA724 before NCT (pre-) and after NCT (post-) were extracted from our database. Survival curves on overall survival (OS) were obtained by Kaplan-Meier method, and differences were analyzed by log-rank test. Associations between categorical variables were explored by chi-square test or Fisher's exact method. Univariable and multivariate analyses were performed by logistic regression model or Cox proportional hazard regression model.

**Results:** TRG was related to OS (P < 0.001), especially when divided into responders (TRG 0-1) and non-responders (TRG 2-3). Pre-CA724 (p = 0.029) and post-CA199 (p = 0.038) were related to OS. In multivariable analysis, pre-CA724 (p = 0.015) and post-CA199 (p = 0.007) were independent prognostic factors for OS, respectively. The changes (diff-) of all tumor markers were not related to OS. Among the clinical characteristics, pre-CA724 (P = 0.047) and tumor size (P = 0.012) were related to TRG, while pre-CA199 (P = 0.377) and pre-CA125 (P = 0.856) were not. In logistics analysis, pre-CA724 (P = 0.032), tumor size (P = 0.011) and tumor location (P = 0.047) were independent risk factors to pathological response.

**Conclusion:** CA724 was an independent prognostic factor for OS and could be used to predict pathological response.

## Introduction

Gastric cancer (GC) is a common digestive tract malignancy, and third leading cause of death worldwide 1. Neoadjuvant chemotherapy (NCT) followed by surgery has become a recommended treatment for locally advanced gastric cancer, because the results of many clinical trials showed NCT could induce tumor down-staging 2, tumor volume reducing 3, resectability increasing 4, elimination of micrometastases 5, and improving survival of patients 6, 7.

The evaluation of the efficacy of NCT is becoming increasingly important. Pathologically, except for TNM stage, tumor regression grade (TRG) is widely used to assess the reaction of tumor 8. There are many different TRG standards, such as standards comparing the relative relationship between residual tumor and fibrosis 9, standards calculating the proportion of residual tumor in the tumor bed 10, and standards only pay attention to the amount of residual tumor 11. Nevertheless, most studies showed TRG could be used as a predictor for prognosis, especially when grouped into responders and non-responders 12, 13.

TRG plays an important role in the evaluation of effectiveness of neoadjuvant therapy. TRG is related to many pathological characteristics, including ypT 14, ypN 15, histological type 16 and, Lauren classification 17. However, the relationships between TRG and some clinical factors such as tumor markers before treatment are still unclear and it is unknown which groups of patients tend to have a better tumor regression.

In this study, we verified the prognostic significance of TRG, investigated the associations between TRG and some clinical factors, and explored the predictors of TRG.

## Methods

### Patients

The information about patients who had locally advanced gastric adenocarcinoma and received NCT between January 2010 and July 2016 at our institute were identified from our electronic database. The criteria for inclusion were: (1) pathologically proved gastric adenocarcinoma; (2) locally advanced gastric cancer (8th American Joint Committee on Cancer [AJCC] clinical stage II-III); (3) received NCT with or without postoperative treatment; and (4) underwent curative gastrectomy surgery. The exclusion criteria were: (1) received preoperative radiotherapy; (2) gastric remnant cancer or suffering from other malignant tumors; or (3) incomplete information on staging or tumor marker before treatment. Among 3,196 patients, 290 were locally advanced gastric cancer patients who underwent neoadjuvant therapy. 141 had all three serum tumor markers before neoadjuvant therapy and 95 had all three serum tumor markers before and after neoadjuvant therapy.

### Pathological response assessment

The slices or blocks indicating the primary tumor of all patients were retrieved from the biospecimen library of our hospital. Two experienced gastrointestinal pathologists (Y.Z. and D.L.) reviewed all slices respectively without the knowledge of clinicopathological information of patients. Pathological TNM stage was reevaluated in accordance with the eighth edition of the AJCC cancer staging guideline. Pathological response of the primary tumor was assessed according to the CAP system: TRG 0 (No viable cancer cells, i.e. complete response), TRG 1 (Single cells or rare small groups of cancer cells, i.e. near complete response), TRG 2 (Residual cancer with evident tumor regression, but more than single cells or rare small groups of cancer cells, i.e. partial response), and TRG 3 (Extensive residual cancer with no evident tumor regression, i.e. poor or no response). When there was disagreement between pathologists, a consensus would be reached by joint rereview and discussion through a multi-head microscope. Other extracted clinicopathological characteristics were reconfirmed during the evaluation process.

### Measurement of serum tumor markers

The levels of serum tumor markers before neoadjuvant chemotherapy (pre-) were measured within 14 days before initial treatment, and the levels after neoadjuvant (post-) chemotherapy were measured within 14 days before the gastrectomy. The changes of tumor markers (diff-) indicated differences between post- and pre- groups. The cutoff values of CA199, CA125 and CA724 were 37 U/ml, 35 U/ml and 8.2 U/ml, respectively.

### Statistical methods

The relationships of categorical variables were calculated by the chi-square test or Fisher's exact test. Logistic regression analysis was used to identify the factors associated with pathological response. To explore the predictors of the pathological response, only clinical variables were included in the analysis. Cox proportional hazard regression model was used to assess the prognostic risk of clinical variables. Variables with p < 0.05 in the multivariate analysis were considered significant. Survival curves were obtained using the Kaplan-Meier method, and log-rank test was used to compare survival differences. All patients were followed up every three months during the first two years, every six months for the following three years and annually thereafter. Overall survival (OS) was defined as the time from the first day of neoadjuvant therapy to the day of death from any cause or last follow-up day. Data was processed by SPSS ver. 25.0 (IBM Corp., Armonk, NY) and R 3.6.1 software (R Foundation for Statistical Computing, Vienna, Austria).

## Results

### Patient characteristics

The clinical features of 141 patients are shown in **Table 1**, and the pathological characteristics are shown in **Table 2**. There were 97 males (68.8%) and 44 females (31.2%), with age ranging from 33 to 76 years (median 58 years). Most of the tumors were located in the lower third part of the stomach (57.4%), and only 2 (1.4%) were located in the gastroesophageal junction (GEJ). Most patients underwent preoperative chemotherapy with SOX (79.4%), and few patients received FOLFOX (17.0%) and XELOX (3.5%). The median number of NCT cycle was 2 (range from 2 to 4). The median operation interval, the time between the completion of neoadjuvant treatment and surgery, was 32 days, with an interquartile range from 29 to 37 days. The median follow-up time of all patients was 36 months (range from 3 to 81 months).

### Pathological assessment

**Figure 1:**
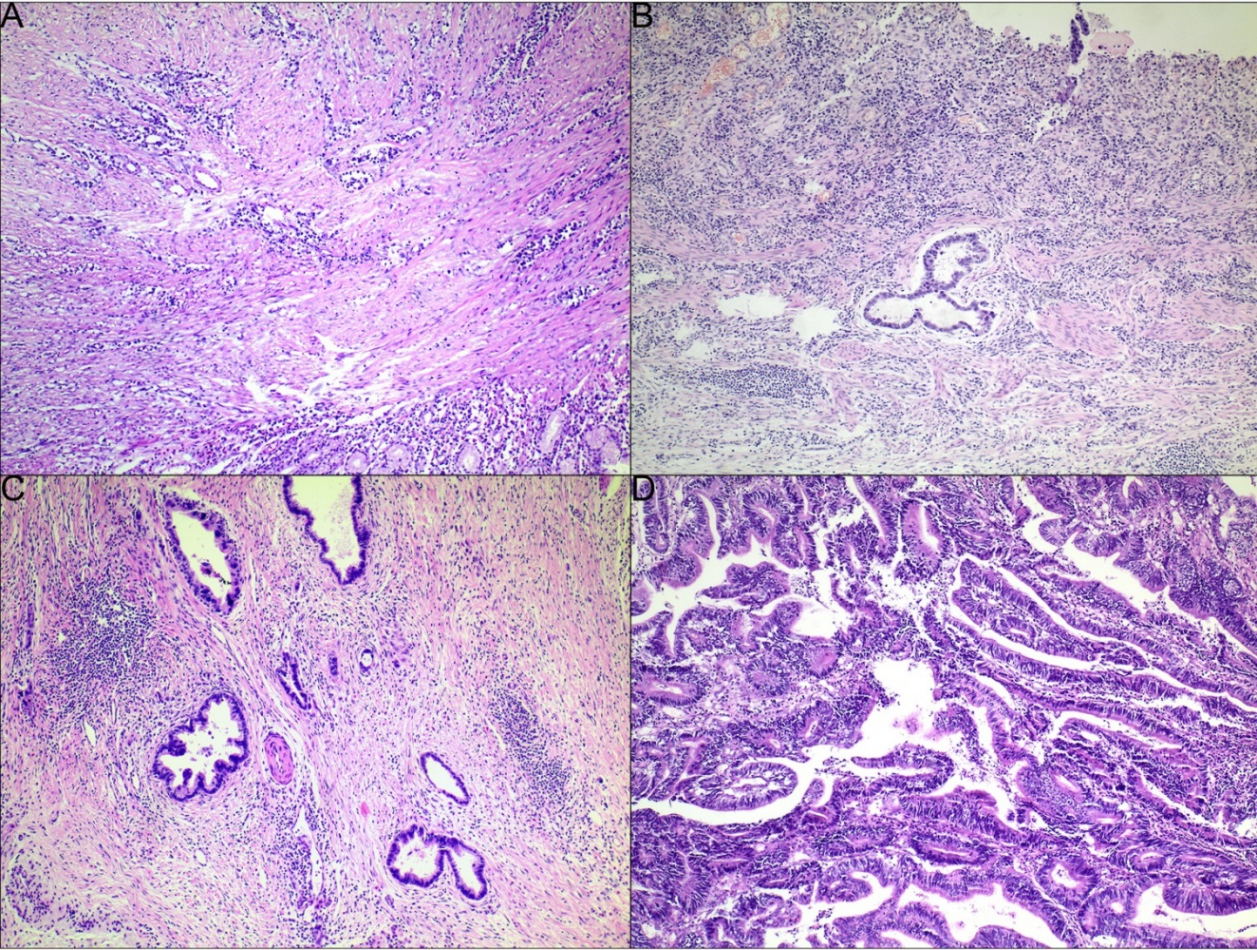
Examples of CAP TRG (A-D): (A) TRG 0, complete tumor regression; (B) TRG 1, single cells or rare small groups of cancer cells; (C) TRG 2, residual cancer with evident tumor regression, but more than single cells or rare small groups of cancer cells; (D) TRG 3, extensive residual cancer with no evident tumor regression.

**Figure 2:**
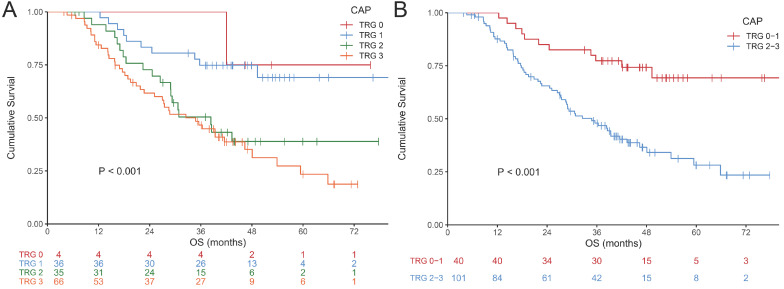
Kaplan-Meier curves for overall survival of CAP TRG. (A) four-tier TRG; (B) pathological response (TRG 0-1 vs 2-3).

The examples of CAP TRG are shown in **Fig. 1**. Totally, 693 slices indicating surgical specimens were reviewed. The median number of reviewed slices was 4, with an interquartile range from 3 to 5. After revaluation, the number of patients was 4, 36, 35 and 66 in the group of TRG 0-3, respectively. There was no significant difference in survival between TRG 0 and TRG 1 (P = 0.775), so these two categories were classified into the responder group. Similarly, no significant difference was found between TRG 2 and TRG 3 (P = 0.383), so these two categories were classified into the non-responder group. The survival curves of CAP TRG were shown in **Fig. 2**.

### Predictive indicators for tumor regression grade

For tumor markers, all included patients have information on three tumor markers before treatment. The numbers of patients who had positive tumor markers were 24, 9 and 34 for pre-CA199, pre-CA125 and pre-CA724, respectively. Patients with high level of pre-CA724 tended to have a worse pathological response (P = 0.047). However, no similar associations were found for pre-CA199 (P = 0.377) and pre-CA125 (P = 0.856) (**Table 1**). The univariable analysis showed that high level of pre-CA724 was associated with poor pathological response (OR = 3.803, P = 0.019), while the pre-CA199 (P = 0.171) and pre-CA125 (P = 0.674) were not relevant to the tumor reaction (**Table 3**). In multivariable analysis, pre-CA724 was an independent risk factor for the pathological response (P = 0.032) (**Table 3**). In addition, 95 patients who also had information on tumor markers after neoadjuvant therapy were analyzed, but CA724 was no longer an independent risk factor (P = 0.150) (data not shown).

For other characteristics, tumor size was associated with TRG. Patients with larger tumor tended to have a worse TRG (P = 0.012) (**Table 1**). In logistic analysis, tumor size did not contribute to a worse pathological response in univariable analysis (P = 0.062), but in multivariable analysis, it became an independent risk factor (P = 0.011) (**Table 3**). It was surprising that tumor location was related to pathological response in the logistic analysis, and tumor in the middle third of the stomach tended to have a better pathological response when compared with tumor located in the gastroesophageal junction and the upper third of the stomach (P < 0.05).

### Prognostic values of serum tumor markers

**Figure 3:**
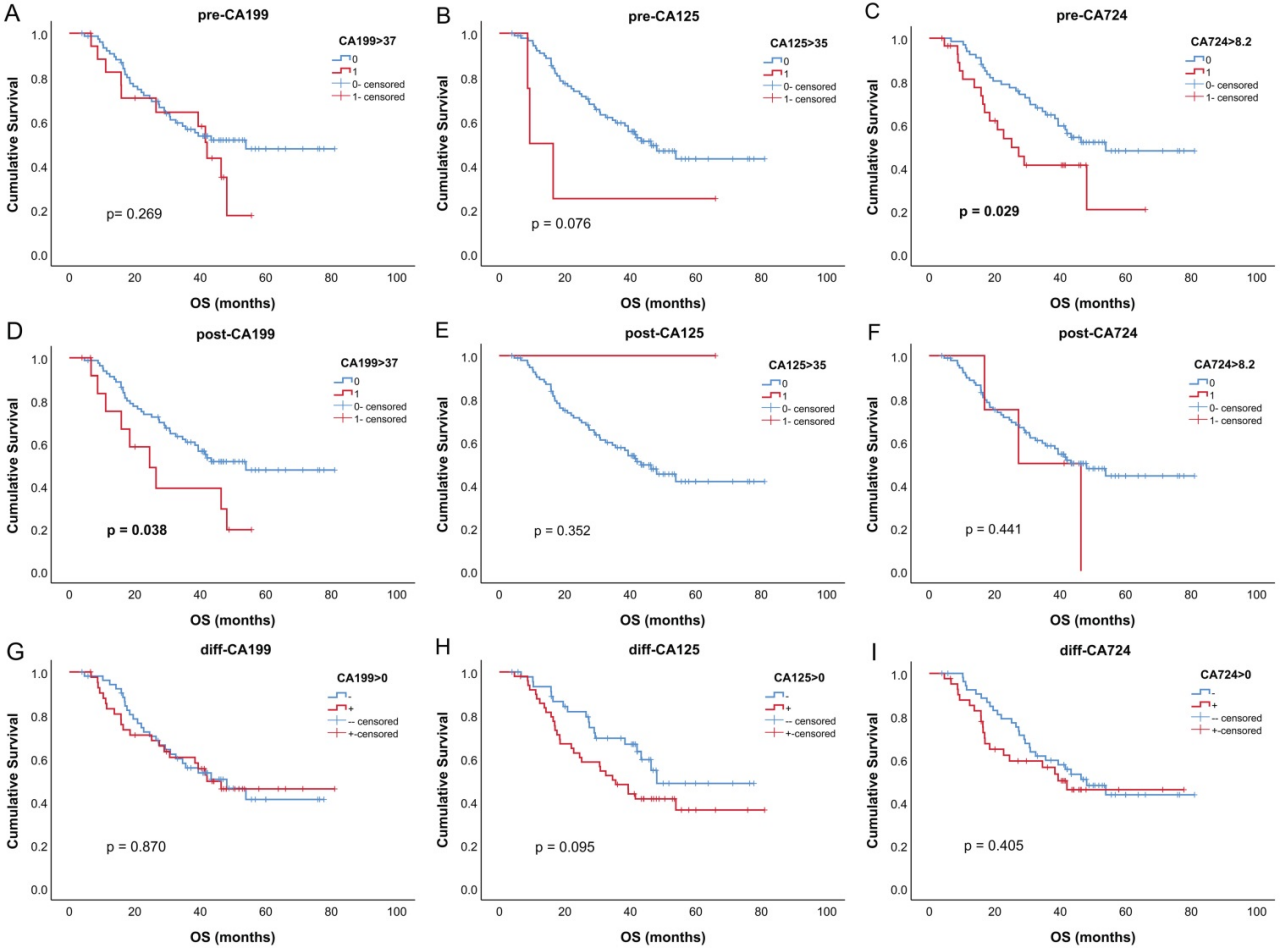
Kaplan-Meier curves for overall survival of tumor markers. (A-C) before neoadjuvant chemotherapy (NCT); (D-F) after NCT; (G-I) change between before and after NCT.

For patients who had all tumor markers before and after neoadjuvant therapy, survival analysis was performed. Survival curves of all tumor markers were shown in **Fig. 3**. Only pre-CA724 (p = 0.029) and post-CA199 (p = 0.038) were related to overall survival. In univariable analysis, pre-CA724 was related to prognosis (p = 0.032), while pre-CA199 (p = 0.272) and pre-CA125 (p = 0.089) were not. In multivariable analysis, pre-CA724 was an independent prognostic factor (p = 0.015) (**Table 4**). However, in multivariable analysis including tumor markers after neoadjuvant therapy, post-CA199 (p = 0.007) was an independent prognostic factor while post-CA724 (p = 0.723) was not (**Table S1**). In multivariable analysis including changes of tumor markers, none of the three tumor markers was related to overall survival (all p > 0.05) (data not shown).

## Discussion

As neoadjuvant therapy has been successfully introduced in the gastrointestinal malignancy, the assessment of efficacy of preoperative therapy is of great importance. Tumor regression grade is a widely used standard to evaluate the effectiveness, but there are many different versions. This study is based on CAP TRG, which is derived from Mandard TRG 9, but a little different 8. Mandard TRG focuses on the relative relationship between residual tumor and fibrosis while CAP TRG merely concentrates on the amount of residual tumor. Compared with Mandard TRG, CAP TRG does not need to distinguish between fibrosis due to tumor regression and fibrosis in the normal area that has never been invaded by tumor. In other words, CAP TRG is easier to carry out and has a better consistency among observers. Nevertheless, this standard still needs to be improved to clearly distinguish different grades by using specific quantities of residual tumor. Although there are some differences between these two TRG standards, these differences will be narrowed when they are divided into responders and non-responders, based on their definitions.

Many researches have proved that responders have a better prognosis than non-responders, so if relationships between TRG and other clinical factors could be revealed, it is possible to predict TRG. Therefore, this study explored the predictors of TRG from characteristics before treatment, and found that CA724 before treatment was an independent risk factor to pathological reaction. In this aspect, only a few researches concentrated on the association of tumor markers and neoadjuvant therapy in gastric cancer. Sun et al. 18 found that CA199 and CA724 were related to overall survival in patients who underwent neoadjuvant chemotherapy, but these two tumor markers were not related to pathological response. This difference might be because their study was based on Backer TRG, and they used a different cutoff value on CA724. Zou et al. 19 found that the decline of CA724 was related to the effectiveness of neoadjuvant therapy. The difference was that they used RECIST criteria rather than TRG. In our another article 20, we found that CA724 before treatment and CA724 after treatment were both independent predictive factors for overall survival. However, in that article, TRG was not mentioned. In addition, tumor size and tumor location showed the predictive power to TRG. However, because of lack of evidence, more researches based on larger sample size are needed to verify this result.

This study also found TRG was related to various pathological factors. This conclusion is similar to other studies. In other studies, TRG was found to be associated with ypT stage 14, 15, 21, ypN stage 22, 23, histological type 16, Lauren classification 17, differentiation grade 15, 23, lymphovascular invasion 21, 23 and nervous invasion 24. Nevertheless, these characteristics after treatment could not be used as a predictor to TRG because these factors and TRG are all obtained at the stage of pathological evaluation.

There are some limitations in this study. This study is retrospective and conducted at a single institution, which means there might be a potential selection bias. This study is based on a relatively small sample size, so a subgroup analysis was not performed. This study used normal cutoff values of tumor markers; however, it is unknown that whether the optimal cutoff values would change because of preoperative treatment. To determine the optimal cutoff values of tumor markers, further researches based on larger sample size are needed. Nevertheless, our study was based on a specific group of patients, explored predictors of TRG from clinical characteristics, and found a relationship between tumor markers and TRG.

In conclusion, CA724 before NCT was an independent prognostic factor for prognosis and could be used as a predictor for TRG in locally advanced gastric cancer patients who underwent neoadjuvant therapy and curative operation.

## Supplementary Material

Supplementary table.

## Figures and Tables

**Table 1:** Patients' characteristics according to CAP TRG

Variable	TRG 0-1 (n=40)	TRG 2 (n=35)	TRG 3 (n=66)	P	No. (%)
**Gender**				0.308	
Male	24 (24.7)	24 (24.7)	49 (50.5)		97 (68.8)
Female	16 (36.4)	11 (25.0)	17 (38.6)		44 (31.2)
**Age (yr)**				0.555	
<65	32 (29.1)	25 (22.7)	53 (48.2)		110 (78.0)
≥65	8 (25.8)	10 (32.3)	13 (41.9)		31 (22.0)
**Tumor location**				0.074	
UGEJ	4 (19.4)	7 (33.3)	10 (47.6)		21 (14.9)
Middle third	13 (54.2)	5 (20.8)	6 (25.0)		24 (17.0)
Lower third	18 (22.2)	20 (24.7)	43 (53.1)		81 (57.4)
Diffuse	5 (33.3)	3 (20.0)	7 (46.7)		15 (10.6)
**Tumor size (cm)**				**0.012**	
<5	19 (38.0)	16 (32.0)	15 (30.0)		50 (35.5)
≥5	21 (23.1)	19 (20.9)	51 (56.0)		91 (64.5)
**cT**				0.504	
2-3	2 (20.0)	4 (40.0)	4 (40.0)		10 (7.1)
4	38 (29.0)	31 (23.7)	62 (47.3)		131 (92.9)
**cN**				0.282	
-	16 (33.3)	14 (29.2)	18 (37.5)		48 (34.0)
+	24 (25.8)	21 (22.6)	48 (51.6)		93 (66.0)
**NCT**				0.101	
FOLFOX	4 (16.7)	3 (12.5)	17 (70.8)		24 (17.0)
SOX	34 (30.4)	30 (26.8)	48 (42.9)		112 (79.4)
XELOX	2 (40.0)	2 (40.0)	1 (20.0)		5 (3.5)
**NCT cycles**				0.846	
2	22 (25.9)	22 (25.9)	41 (48.2)		85 (60.3)
3	8 (38.1)	5 (23.8)	8 (38.1)		21 (14.9)
4	10 (28.6)	8 (22.9)	17 (48.6)		35 (24.8)
**pre-CA199**				0.377	
-	36 (30.8)	28 (23.9)	53 (45.3)		117 (83.0)
+	4 (16.7)	7 (29.2)	13 (54.2)		24 (17.0)
**pre-CA125**				0.856	
-	38 (28.8)	33 (25.0)	61 (46.2)		132 (93.6)
+	2 (22.2)	2 (22.2)	5 (55.6)		9 (6.4)
**pre-CA724**				**0.047**	
-	36 (33.6)	25 (23.4)	46 (43.0)		107 (75.9)
+	4 (11.8)	10 (29.4)	20 (58.8)		34 (24.1)

Note: TRG, tumor regression grade; UGEJ, upper third and gastroesophageal junction.

**Table 2:** Patients' characteristics according to CAP TRG

Variable	TRG 0-1 (n=40)	TRG 2 (n=35)	TRG 3 (n=66)	P	No. (%)
**ypT**				**<0.001**	
0	4 (100.0)	0 (0.0)	0 (0.0)		4 (2.6)
1-2	20 (87.0)	1 (4.3)	2 (8.7)		23 (16.3)
3-4	16 (14.0)	34 (29.8)	64 (56.1)		114 (80.9)
**ypN**				**<0.001**	
0	23 (53.5)	9 (20.9)	11 (25.6)		43 (30.5)
1	9 (32.1)	8 (28.6)	11 (39.3)		28 (19.9)
2	6 (15.4)	9 (23.1)	24 (61.5)		39 (27.7)
3	2 (6.5)	9 (29.0)	20 (64.5)		31 (22.0)
**ypTNM**				**<0.001**	
1-2	32 (59.3)	10 (18.5)	12 (22.2)		54 (38.3)
3	8 (9.2)	25 (28.7)	54 (62.1)		87 (61.7)
**Histological type**				**<0.001**	
Adenocarcinoma	34 (38.6)	24 (27.3)	30 (34.1)		88 (62.4)
Poorly cohesive carcinoma	6 (11.3)	11 (20.8)	36 (67.9)		53 (37.6)
**Lauren classification**				**0.037**	
Intestinal	27 (38.0)	15 (21.1)	29 (40.8)		71 (50.4)
Diffuse or mixed	13 (18.6)	20 (28.6)	37 (52.9)		70 (49.6)
**Grade of differentiation**				**<0.001**	
Well	21 (58.3)	5 (13.9)	10 (27.8)		36 (25.5)
Moderate or poor	19 (18.1)	30 (28.6)	56 (53.3)		105 (74.5)
**Vascular or lymphatic invasion**				**0.019**	
No	35 (35.0)	24 (24.0)	41 (41.0)		100 (70.9)
Yes	5 (12.2)	11 (26.8)	25 (61.0)		41 (29.1)
**Nervous invasion**				**<0.001**	
No	40 (39.2)	18 (17.6)	44 (43.1)		102 (72.3)
Yes	0 (0.0)	17 (43.6)	22 (56.4)		39 (27.7)
**Adjuvant treatment**				0.319	
No	8 (42.1)	3 (15.8)	9 (47.4)		19 (13.5)
Yes	32 (26.2)	32 (26.2)	58 (47.5)		122 (86.5)

Note: TRG, tumor regression grade; UGEJ, upper third and gastroesophageal junction.

**Table 3:** Logistic analysis for pathological response

Variable	Univariable analysis		Multivariable analysis	
	OR (95%CI)	P	OR (95%CI)	P
Gender (Female)	0.575 (0.267, 1.240)	0.158	0.618 (0.241, 1.583)	0.316
Age (≥65yr)	1.179 (0.478, 2.912)	0.555	0.991 (0.338, 2.902)	0.987
**Tumor location**		**0.022**		**0.047**
UGEJ	1		1	
Middle third	0.199 (0.051, 0.770)	**0.019**	0.132 (0.028, 0.615)	**0.010**
Lower third	0.824 (0.246, 2.758)	0.753	0.477 (0.125, 1.821)	0.279
Diffuse	0.471 (0.102, 2.172)	0.334	0.195 (0.034, 1.125)	0.068
**Tumor size (≥5cm)**	2.043 (0.964, 4.329)	0.062	3.433 (1.323, 8.913)	**0.011**
cT (4)	0.612 (0.124, 3.015)	0.546	0.467 (0.075, 2.919)	0.416
cN (+)	1.437 (0.673, 3.070)	0.349	1.413 (0.578, 3.454)	0.449
**NCT**		0.352		0.455
FOLFOX	1		1	
SOX	0.459 (0.146, 1.444)	0.183	0.545 (0.149, 1.994)	0.359
XELOX	0.300 (0.037, 2.417)	0.258	0.244 (0.024, 2.498)	0.235
**NCT cycles**		0.543		0.737
2	1		1	
3	0.567 (0.208, 1.551)	0.269	0.654 (0.189, 2.257)	0.501
4	0.873 (0.362, 2.104)	0.762	0.739 (0.259, 2.103)	0.570
CA199 (+)	2.222 (0.709, 6.970)	0.171	1.533 (0.431, 5.449)	0.509
CA125 (+)	1.415 (0.281, 7.121)	0.674	1.059 (0.157, 7.148)	0.953
CA724 (+)	3.803 (1.244, 11.628)	**0.019**	4.033 (1.128, 14.427)	**0.032**

Note: UGEJ, upper third and gastroesophageal junction.

**Table 4:** Cox proportional hazard regression model for overall survival before neoadjuvant therapy

Variable	Univariable analysis		Multivariable analysis	
	OR (95%CI)	P	OR (95%CI)	P
Gender (Female)	1.460 (0.757, 2.817)	0.259	0.734 (0.364, 1.480)	0.388
Age (≥65yr)	1.268 (0.706, 2.275)	0.427	1.012 (0.481, 2.130)	0.976
**Tumor location**		**0.037**		**0.008**
Middle third	1		1	
UGEJ	2.030 (0.523, 7.874)	0.306	2.366 (0.545, 10.270)	0.250
Lower third	2.161 (0.654, 7.141)	0.206	4.263 (0.932, 19.494)	0.062
Diffuse	5.186 (1.415, 19.012)	**0.013**	9.427 (2.161, 41.125)	**0.003**
**Tumor size (≥5cm)**	2.162 (1.137, 4.110)	**0.019**	2.307 (1.093, 4.871)	**0.028**
cT (4)	0.318 (0.123, 0.817)	**0.017**	0.300 (0.098, 0.918)	**0.035**
cN (+)	1.494 (0.809, 2.760)	0.200	1.472 (0.733, 2.957)	0.277
**NCT**		0.589		0.069
FOLFOX	1		1	
SOX	1.628 (0.644, 4.117)	0.303	3.642 (1.218, 10.890)	**0.021**
XELOX	*	0.979	*	**
**NCT cycles**		0.642		**0.008**
2	1		1	
3	0.900 (0.437, 1.855)	0.775	1.309 (0.531, 3.228)	0.559
4	0.697 (0.329, 1.479)	0.347	0.283 (0.110, 0.724)	**0.008**
pre-CA199 (+)	1.461 (0.743, 2.875)	0.272	2.011 (0.887, 4.563)	0.095
pre-CA125 (+)	2.776 (0.856, 9.004)	0.089	6.012 (1.429, 25.284)	**0.014**
pre-CA724 (+)	1.949 (1.059, 3.586)	**0.032**	2.935 (1.232, 6.991)	**0.015**

Note: UGEJ, upper third and gastroesophageal junction; *too small to record; **too large to record.

## Abbreviations

GC: gastric cancer
NCT: neoadjuvant chemotherapy
TRG: tumor regression grade
OS: overall survival
AJCC: American Joint Committee on Cancer
CA199: carbohydrate antigen 19-9
CA125: cancer antigen 125
CA724: carbohydrate antigen 72-4
GEJ: gastroesophageal junction
ypTNM: post-neoadjuvant therapy tumor-node-metastasis stage
