# Supplementary material for: CA724 Predicts Tumor Regression Grade in Locally Advanced Gastric Cancer Patients with Neoadjuvant Chemotherapy
Source: J Cancer. 2021 Sep 3;12(21):6465–72. doi: 10.7150/jca.60694 (PMC8489128; doi:10.7150/jca.60694)
Supplement: Supplementary file 1 — Supplementary table. [file jcav12p6465s1.pdf]

Table S1 Cox proportional hazard regression model for overall survival after neoadjuvant therapy

	Univariable analysis		Multivariable analysis	
Variable	OR (95%CI)	P	OR (95%CI)	P
Gender (Female)	1.460 (0.757, 2.817)	0.259	1.031 (0.532, 1.996)	0.928
Age (≥65yr)	1.268 (0.706, 2.275)	0.427	0.670 (0.292, 1.536)	0.344
Tumor location		**0.037**		**0.007**
Middle third	1		1	
UGEJ	2.030 (0.523, 7.874)	0.306	0.160 (0.033, 0.765)	**0.022**
Lower third	2.161 (0.654, 7.141)	0.206	1.040 (0.405, 2.668)	0.935
Diffuse	5.186 (1.415, 19.012)	**0.013**	2.672 (0.851, 8.391)	0.092
Tumor size (≥5cm)	2.162 (1.137, 4.110)	**0.019**	2.277 (1.096, 4.731)	**0.027**
cT (4)	0.318 (0.123, 0.817)	**0.017**	0.305 (0.101, 0.927)	**0.036**
cN (+)	1.494 (0.809, 2.760)	0.200	1.760 (0.868, 3.568)	0.117
NCT		0.589		0.093
FOLFOX	1		1	
SOX	1.628 (0.644, 4.117)	0.303	3.327 (1.129, 9.806)	**0.029**
XELOX	*	0.979	*	0.983
NCT cycles		0.642		0.071
2	1		1	
3	0.900 (0.437, 1.855)	0.775	1.524 (0.630, 3.689)	0.350
4	0.697 (0.329, 1.479)	0.347	0.479 (0.201, 1.142)	0.097
post-CA199 (+)	2.129 (1.027, 4.416)	**0.042**	3.552 (1.406, 8.972)	**0.007**
post-CA125 (+)	0.048 (0.000, 727.121)	0.536	*	0.989
post-CA724 (+)	1.579 (0.489, 5.099)	0.445	1.285 (0.320, 5.158)	0.723

Note: UGEJ, upper third and gastroesophageal junction; *too small to record
